# 2-Carb­oxy-1-phenyl­ethanaminium perchlorate

**DOI:** 10.1107/S1600536809016171

**Published:** 2009-05-07

**Authors:** Xiu-Zhi Li, Hui Li, Zhi-Rong Qu

**Affiliations:** a Ordered Matter Science Research Center, College of Chemistry and Chemical Engineering, Southeast University, Nanjing 210096, People’s Republic of China

## Abstract

In the title compound, C_9_H_12_NO_2_^+^·ClO_4_^−^, an intra­molecular N—H⋯O inter­action results in the formation of a six-membered ring having a twisted chair conformation. In the crystal structure, inter­molecular O—H⋯O, N—H⋯O and C—H⋯O inter­actions link the mol­ecules into a network. A weak C—H⋯π inter­action is also found.

## Related literature

There has been an increased inter­est in the enanti­omeric preparation of β-amino acids as precursors for the synthesis of novel biologically active compounds, see: Arki *et al.* (2004 ▶); Cohen *et al.* (2002 ▶); Zeller *et al.* (1965 ▶). For bond-length data, see: Allen *et al.* (1987 ▶).
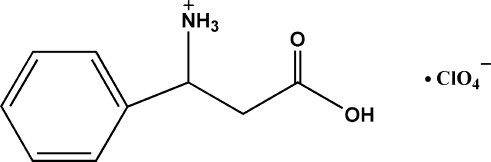


## Experimental

### 

#### Crystal data

C_9_H_12_NO_2_^+^·ClO_4_^−^
*M* *_r_* = 265.65
Orthorhombic, 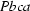

*a* = 6.6583 (13) Å
*b* = 13.826 (3) Å
*c* = 24.300 (5) Å
*V* = 2237.0 (8) Å^3^
*Z* = 8
Mo *K*α radiation
μ = 0.36 mm^−1^
*T* = 294 K
0.45 × 0.35 × 0.12 mm

#### Data collection

Rigaku SCXmini diffractometer
Absorption correction: multi-scan (Blessing, 1995 ▶) *T*_min_ = 0.863, *T*_max_ = 0.957
21012 measured reflections
2560 independent reflections
1966 reflections with *I* > 2σ(*I*)
*R*_int_ = 0.058

#### Refinement

*R*[*F* ^2^ > 2σ(*F* ^2^)] = 0.053
*wR*(*F* ^2^) = 0.110
*S* = 1.10
2560 reflections
156 parameters
H-atom parameters constrained
Δρ_max_ = 0.27 e Å^−3^
Δρ_min_ = −0.37 e Å^−3^

### 

Data collection: *CrystalClear* (Rigaku/MSC, 2005 ▶); cell refinement: *CrystalClear*; data reduction: *CrystalClear*; program(s) used to solve structure: *SHELXS97* (Sheldrick, 2008 ▶); program(s) used to refine structure: *SHELXL97* (Sheldrick, 2008 ▶); molecular graphics: *PLATON* (Spek, 2009 ▶); software used to prepare material for publication: *SHELXL97*.

## Supplementary Material

Crystal structure: contains datablocks I, global. DOI: 10.1107/S1600536809016171/hk2675sup1.cif

Structure factors: contains datablocks I. DOI: 10.1107/S1600536809016171/hk2675Isup2.hkl

Additional supplementary materials:  crystallographic information; 3D view; checkCIF report

## Figures and Tables

**Table 1:** Hydrogen-bond geometry (Å, °)

*D*—H⋯*A*	*D*—H	H⋯*A*	*D*⋯*A*	*D*—H⋯*A*
N1—H1*B*⋯O2	0.89	2.13	2.773 (3)	128
C9—H9⋯O5^i^	0.93	2.56	3.409 (3)	152
C3—H3⋯O5^i^	0.98	2.57	3.370 (3)	139
C3—H3⋯O2^ii^	0.98	2.58	3.286 (3)	129
N1—H1*C*⋯O3^iii^	0.89	2.05	2.892 (3)	158
N1—H1*B*⋯O3^iv^	0.89	2.28	3.046 (3)	144
N1—H1*A*⋯O6^v^	0.89	2.13	2.979 (3)	159
O1—H1⋯O2^vi^	0.82	2.41	3.046 (2)	135
O1—H1⋯O4^vii^	0.82	2.35	3.048 (3)	143
C8—H8⋯*Cg*1^viii^	0.93	2.79	3.688 (3)	162

Symmetry codes: (i) 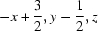; (ii) 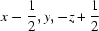; (iii) 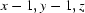; (iv) 
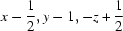
; (v) 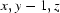; (vi) 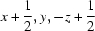; (vii) 
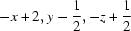
; (viii) 
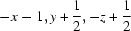
. *Cg*1 is the centroid of the C4–C9 ring.

**Fig. 1.**
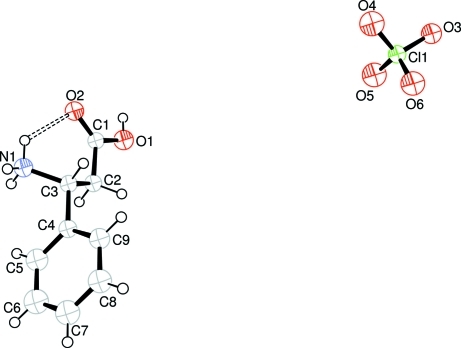
The molecular structure of the title molecule, with the atom-numbering scheme. Hydrogen bond is shown as dashed line.

**Fig. 2.**
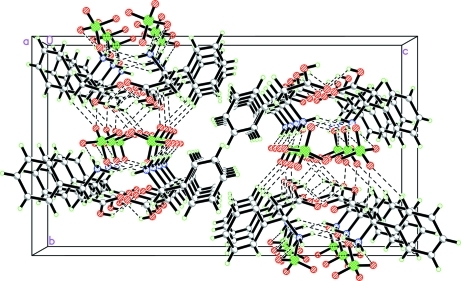
A partial packing diagram of the title compound. Hydrogen bonds are shown as dashed lines.

## supplementary crystallographic information

### Comment

β-Amino acids are important molecules, due to their pharmacological properties. Recently, there has been an increased interest in the enantiomeric preparations of β-amino acids, as precursors for the synthesis of novel biologically active compounds (Arki *et al.*, 2004; Cohen *et al.*, 2002; Zeller *et al.*, 1965). We report herein the crystal structure of the title compound.

The asymmetric unit of the title compound contains one cation and one anion (Fig.1 ), in which the bond lengths (Allen *et al.*,  1987) and angles are within normal ranges. Ring A (C4-C9) is, of course, planar. Intramolecular N-H···O interaction results in the formation of a six-membered ring B (O2/N1/C1-C3/H1B) having twisted conformation.

In the crystal structure, intermolecular O-H···O, N-H···O and C-H···O interactions (Table 1) link the molecules into a network (Fig. 2), in which they may be effective in the stabilization of the structure. There also exists a weak C—H···π interaction (Table 1).

### Experimental

Under nitrogen protection, benzaldehyde (3.18 g, 30 mmol), malonic acid (5.00 g, 48 mmol) and ammonium acetate (6.00 g, 78 mmol) were added into a flask and refluxed for 10 h to yield a colorless precipitate. The crude product was obtained after filtration, then it was dissolved in ethanol/ perchloric acid (1:1), after slowly evaporating over a period of 4 d, colorless prism crystals of the title compound suitable for X-ray analysis were isolated.

### Refinement

H atoms were positioned geometrically with O-H = 0.82 Å (for OH), N-H = 0.89 Å (for NH_3_), C-H = 0.93, 0.98 and 0.97 Å, for aromatic, methine and methylene H atoms, respectively, and constrained to ride on their parent atoms, with U_iso_(H) = xU_eq_(C,O,N), where x = 1.5 for OH and NH_3_ H and x = 1.2 for all other H atoms.

### Crystal data

**Table:**

C_9_H_12_NO_2_^+^·ClO_4_^−^	*F*(000) = 1104
*M**_r_* = 265.65	*D*_x_ = 1.578 Mg m^−^^3^
Orthorhombic, *P**b**c**a*	Mo *K*α radiation, λ = 0.71073 Å
Hall symbol: -P 2ac 2ab	Cell parameters from 1979 reflections
*a* = 6.6583 (13) Å	θ = 3.1–27.5°
*b* = 13.826 (3) Å	µ = 0.36 mm^−^^1^
*c* = 24.300 (5) Å	*T* = 294 K
*V* = 2237.0 (8) Å^3^	Prism, colorless
*Z* = 8	0.45 × 0.35 × 0.12 mm

### Data collection

**Table:**

Rigaku SCXmini diffractometer	2560 independent reflections
Radiation source: fine-focus sealed tube	1966 reflections with *I* > 2σ(*I*)
graphite	*R*_int_ = 0.058
CCD_Profile_fitting scans	θ_max_ = 27.5°, θ_min_ = 3.1°
Absorption correction: multi-scan (Blessing, 1995)	*h* = −8→8
*T*_min_ = 0.863, *T*_max_ = 0.957	*k* = −17→17
21012 measured reflections	*l* = −31→31

### Refinement

**Table:**

Refinement on *F*^2^	Primary atom site location: structure-invariant direct methods
Least-squares matrix: full	Secondary atom site location: difference Fourier map
*R*[*F*^2^ > 2σ(*F*^2^)] = 0.053	Hydrogen site location: inferred from neighbouring sites
*wR*(*F*^2^) = 0.110	H-atom parameters constrained
*S* = 1.10	*w* = 1/[σ^2^(*F*_o_^2^) + (0.0351*P*)^2^ + 1.6729*P*] where *P* = (*F*_o_^2^ + 2*F*_c_^2^)/3
2560 reflections	(Δ/σ)_max_ < 0.001
156 parameters	Δρ_max_ = 0.27 e Å^−^^3^
0 restraints	Δρ_min_ = −0.37 e Å^−^^3^

### Special details

**Table:**

Geometry. All e.s.d.'s (except the e.s.d. in the dihedral angle between two l.s. planes) are estimated using the full covariance matrix. The cell e.s.d.'s are taken into account individually in the estimation of e.s.d.'s in distances, angles and torsion angles; correlations between e.s.d.'s in cell parameters are only used when they are defined by crystal symmetry. An approximate (isotropic) treatment of cell e.s.d.'s is used for estimating e.s.d.'s involving l.s. planes.
Refinement. Refinement of *F*^2^ against ALL reflections. The weighted *R*-factor *wR* and goodness of fit *S* are based on *F*^2^, conventional *R*-factors *R* are based on *F*, with *F* set to zero for negative *F*^2^. The threshold expression of *F*^2^ > σ(*F*^2^) is used only for calculating *R*-factors(gt) *etc*. and is not relevant to the choice of reflections for refinement. *R*-factors based on *F*^2^ are statistically about twice as large as those based on *F*, and *R*- factors based on ALL data will be even larger.

### Fractional atomic coordinates and isotropic or equivalent isotropic displacement parameters (Å^2^)

**Table:**

	*x*	*y*	*z*	*U*_iso_*/*U*_eq_	
Cl1	0.99041 (8)	0.97917 (4)	0.32015 (2)	0.03337 (17)	
O1	0.9660 (3)	0.30715 (14)	0.32107 (7)	0.0428 (5)	
H1	1.0262	0.3182	0.2923	0.064*	
O2	0.7570 (2)	0.22973 (13)	0.26501 (6)	0.0365 (4)	
O3	1.0688 (3)	1.07288 (12)	0.30762 (7)	0.0439 (5)	
O4	0.9012 (3)	0.94103 (16)	0.27168 (8)	0.0630 (6)	
O5	1.1483 (3)	0.91952 (15)	0.33824 (10)	0.0649 (6)	
O6	0.8413 (3)	0.98786 (15)	0.36203 (8)	0.0584 (6)	
N1	0.4988 (3)	0.09957 (16)	0.31532 (7)	0.0349 (5)	
H1A	0.5772	0.0550	0.3307	0.052*	
H1B	0.5491	0.1165	0.2828	0.052*	
H1C	0.3759	0.0756	0.3107	0.052*	
C1	0.8076 (3)	0.25365 (17)	0.31064 (9)	0.0289 (5)	
C2	0.6999 (3)	0.22487 (17)	0.36217 (9)	0.0306 (5)	
H2A	0.6911	0.2806	0.3863	0.037*	
H2B	0.7778	0.1756	0.3810	0.037*	
C3	0.4889 (3)	0.18613 (17)	0.35177 (9)	0.0289 (5)	
H3	0.4139	0.2364	0.3321	0.035*	
C4	0.3749 (3)	0.16333 (17)	0.40392 (9)	0.0310 (5)	
C5	0.3897 (4)	0.0760 (2)	0.43014 (10)	0.0464 (7)	
H5	0.4766	0.0289	0.4167	0.056*	
C6	0.2752 (5)	0.0576 (2)	0.47670 (12)	0.0565 (8)	
H6	0.2840	−0.0023	0.4940	0.068*	
C7	0.1499 (4)	0.1267 (2)	0.49722 (11)	0.0537 (8)	
H7	0.0739	0.1143	0.5286	0.064*	
C8	0.1367 (4)	0.2138 (2)	0.47155 (11)	0.0493 (7)	
H8	0.0524	0.2613	0.4857	0.059*	
C9	0.2472 (4)	0.2322 (2)	0.42480 (9)	0.0390 (6)	
H9	0.2352	0.2916	0.4072	0.047*	

### Atomic displacement parameters (Å^2^)

**Table:**

	*U*^11^	*U*^22^	*U*^33^	*U*^12^	*U*^13^	*U*^23^
Cl1	0.0277 (3)	0.0306 (3)	0.0418 (3)	−0.0002 (2)	0.0000 (2)	0.0054 (3)
O1	0.0336 (10)	0.0582 (12)	0.0365 (9)	−0.0188 (8)	0.0033 (8)	−0.0024 (9)
O2	0.0335 (9)	0.0489 (10)	0.0270 (8)	−0.0081 (8)	0.0018 (7)	−0.0040 (7)
O3	0.0422 (10)	0.0332 (10)	0.0564 (11)	−0.0080 (8)	−0.0014 (9)	0.0095 (8)
O4	0.0594 (13)	0.0684 (14)	0.0612 (13)	−0.0217 (11)	−0.0103 (11)	−0.0133 (11)
O5	0.0553 (13)	0.0554 (13)	0.0841 (15)	0.0219 (11)	−0.0044 (12)	0.0233 (11)
O6	0.0517 (12)	0.0644 (13)	0.0591 (12)	0.0037 (10)	0.0210 (10)	0.0119 (11)
N1	0.0307 (11)	0.0439 (12)	0.0300 (10)	−0.0085 (9)	0.0019 (9)	−0.0045 (9)
C1	0.0242 (11)	0.0291 (12)	0.0333 (12)	0.0002 (9)	0.0011 (9)	−0.0020 (10)
C2	0.0296 (12)	0.0359 (13)	0.0262 (11)	−0.0056 (10)	−0.0013 (9)	−0.0024 (10)
C3	0.0253 (11)	0.0355 (13)	0.0259 (11)	0.0009 (10)	0.0013 (9)	−0.0003 (9)
C4	0.0238 (11)	0.0435 (14)	0.0256 (11)	−0.0039 (10)	−0.0005 (9)	0.0008 (10)
C5	0.0485 (16)	0.0501 (16)	0.0406 (14)	0.0037 (13)	0.0071 (13)	0.0065 (13)
C6	0.066 (2)	0.0596 (19)	0.0435 (16)	−0.0121 (16)	0.0047 (15)	0.0180 (14)
C7	0.0425 (16)	0.085 (2)	0.0339 (14)	−0.0167 (16)	0.0088 (12)	0.0037 (15)
C8	0.0388 (15)	0.073 (2)	0.0363 (14)	0.0045 (14)	0.0094 (12)	−0.0057 (14)
C9	0.0323 (12)	0.0520 (16)	0.0326 (12)	0.0016 (11)	0.0003 (11)	0.0012 (12)

### Geometric parameters (Å, °)

**Table:**

Cl1—O5	1.4067 (19)	C3—N1	1.490 (3)
Cl1—O4	1.421 (2)	C3—C4	1.510 (3)
Cl1—O6	1.4269 (19)	C3—H3	0.9800
Cl1—O3	1.4297 (18)	C4—C5	1.369 (3)
O1—H1	0.8200	C4—C9	1.373 (3)
N1—H1A	0.8900	C5—C6	1.388 (4)
N1—H1B	0.8900	C5—H5	0.9300
N1—H1C	0.8900	C6—C7	1.363 (4)
C1—O2	1.205 (3)	C6—H6	0.9300
C1—O1	1.313 (3)	C7—C8	1.359 (4)
C1—C2	1.497 (3)	C7—H7	0.9300
C2—C3	1.524 (3)	C8—C9	1.377 (3)
C2—H2A	0.9700	C8—H8	0.9300
C2—H2B	0.9700	C9—H9	0.9300
			
O5—Cl1—O4	110.73 (15)	N1—C3—C2	109.89 (18)
O5—Cl1—O6	110.28 (13)	C4—C3—C2	113.42 (18)
O4—Cl1—O6	109.36 (13)	N1—C3—H3	107.5
O5—Cl1—O3	108.97 (12)	C4—C3—H3	107.5
O4—Cl1—O3	108.21 (12)	C2—C3—H3	107.5
O6—Cl1—O3	109.25 (12)	C5—C4—C9	119.0 (2)
C1—O1—H1	109.5	C5—C4—C3	122.6 (2)
C3—N1—H1A	109.5	C9—C4—C3	118.5 (2)
C3—N1—H1B	109.5	C4—C5—C6	120.1 (3)
C3—N1—H1C	109.5	C4—C5—H5	120.0
H1A—N1—H1B	109.5	C6—C5—H5	120.0
H1A—N1—H1C	109.5	C7—C6—C5	120.4 (3)
H1B—N1—H1C	109.5	C7—C6—H6	119.8
O2—C1—O1	123.8 (2)	C5—C6—H6	119.8
O2—C1—C2	124.2 (2)	C8—C7—C6	119.5 (3)
O1—C1—C2	111.91 (19)	C8—C7—H7	120.2
C1—C2—C3	113.35 (18)	C6—C7—H7	120.2
C1—C2—H2A	108.9	C7—C8—C9	120.5 (3)
C3—C2—H2A	108.9	C7—C8—H8	119.7
C1—C2—H2B	108.9	C9—C8—H8	119.7
C3—C2—H2B	108.9	C4—C9—C8	120.5 (3)
H2A—C2—H2B	107.7	C4—C9—H9	119.7
N1—C3—C4	110.68 (19)	C8—C9—H9	119.7

### Hydrogen-bond geometry (Å, °)

**Table:**

*D*—H···*A*	*D*—H	H···*A*	*D*···*A*	*D*—H···*A*
N1—H1B···O2	0.89	2.13	2.773 (3)	128
C9—H9···O5^i^	0.93	2.56	3.409 (3)	152
C3—H3···O5^i^	0.98	2.57	3.370 (3)	139
C3—H3···O2^ii^	0.98	2.58	3.286 (3)	129
N1—H1C···O3^iii^	0.89	2.05	2.892 (3)	158
N1—H1B···O3^iv^	0.89	2.28	3.046 (3)	144
N1—H1A···O6^v^	0.89	2.13	2.979 (3)	159
O1—H1···O2^vi^	0.82	2.41	3.046 (2)	135
O1—H1···O4^vii^	0.82	2.35	3.048 (3)	143
C8—H8···Cg1^viii^	0.93	2.79	3.688 (3)	162

Symmetry codes: (i) −*x*+3/2, *y*−1/2, *z*; (ii) *x*−1/2, *y*, −*z*+1/2; (iii) *x*−1, *y*−1, *z*; (iv) *x*−1/2, *y*−1, −*z*+1/2; (v) *x*, *y*−1, *z*; (vi) *x*+1/2, *y*, −*z*+1/2; (vii) −*x*+2, *y*−1/2, −*z*+1/2; (viii) −*x*−1, *y*+1/2, −*z*+1/2.
